# Cytomegalovirus pneumonia in an immunosuppressed sarcoidosis patient; a rare case of cytomegalovirus infection in a sarcoidosis patient

**DOI:** 10.22088/cjim.12.0.404

**Published:** 2021

**Authors:** Mahnaz Mozdourian, Rozita khodashahi

**Affiliations:** 1 Lung Diseases Research Center, Mashhad University of Medical Sciences, Mashhad, Iran; 2 Department of Infectious Diseases and Tropical Medicine, Faculty of Medicine, Mashhad University of Medical Sciences, Mashhad, Iran

**Keywords:** Sarcoidosis, Cytomegalovirus, Pneumonia

## Abstract

**Background:**

Sarcoidosis is a multisystemic granulomatosis disease that is mostly treated with immunosuppressive regimens. Studies demonstrated that these patients are prone to develop various infections. However, some infections including viral severe pneumonia is rare complications in sarcoidosis patients. In the present report, we described for cytomegalovirus (CMV) pneumonia in a female patient with sarcoidosis which has been successfully managed by ganciclovir.

**Case Presentation:**

Herein, we present a known case of sarcoidosis admitted to the emergency department because of fever, dyspnea, and productive cough. The patient was receiving prednisolone and methotrexate for months. The primary chest x-ray imaging revealed bilateral infiltration, especially in the upper lobes and hilar lymphadenopathy. The lung high resolution computed tomography showed a bilateral diffuse nodular pattern. After 72 hours of antimicrobial treatment, the fever was still present and the patient became a candidate for fiberoptic bronchoscopy. The gram staining of the bronchial fluid, polymerase chain reaction for tuberculosis, and PCP was also unremarkable. However, the PCR-CMV was positive. The quantitative PCR for CMV form blood sample was taken and the result came back as 3.6*10^3^. With the impression of CMV pneumonia, a daily dose of 5mg of ganciclovir was prescribed. After 3 weeks of receiving 5mg/kg of ganciclovir twice daily (600mg daily), clinical symptoms, and dyspnea improved. Also, the radiological findings improved.

**Conclusion:**

In the present report, we demonstrated that sarcoidosis patients’ receiving immunosuppressives are prone to develop CMV pneumonia, and fever and dyspnea were the alarm signs of CMV pneumonia is our patient which was successfully managed by ganciclovir.

Sarcoidosis is a granulomatosis disease that is characterized by giant cell granuloma (1). The prevalence of sarcoidosis is reported to be 160 per 100000 individuals (2). Although the exact etiology of this multisystemic disease is not known; however, the overactive immune response to antigens is considered as a possible reason for the development of sarcoidosis (1). Sarcoidosis is usually an afebrile granulomatous disorder and the presence of fever mostly highlights a possible infection or malignancy (3). Treatment of sarcoidosis usually depends on glucocorticoids and cytotoxic drugs (1). Using immunosuppressive drugs affected these patients to develop opportunistic infections (1). Cytomegalovirus (CMV) is a common pathogen causing interstitial pneumonia (4). 

CMV interstitial pneumonia could be a life-threatening infection in immunocompromised patients but is rarely reported in sarcoidosis patients (4). In the present report, we discussed a case of CMV pneumonia a sarcoidosis patient receiving immunosuppressants. 

## Case presentation

**Figure 1:**
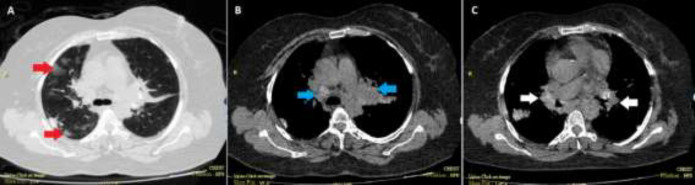
The chest high resolution computed tomography of the patients. A) The red arrows show multiple nodules in the upper lobe of right lung. B) In the aortopulmonary window, paratracheal lymphadenopathies are shown by blue arrows. C) Hilar lymphadenopathies are shown by white arrows

**Figure 2:**
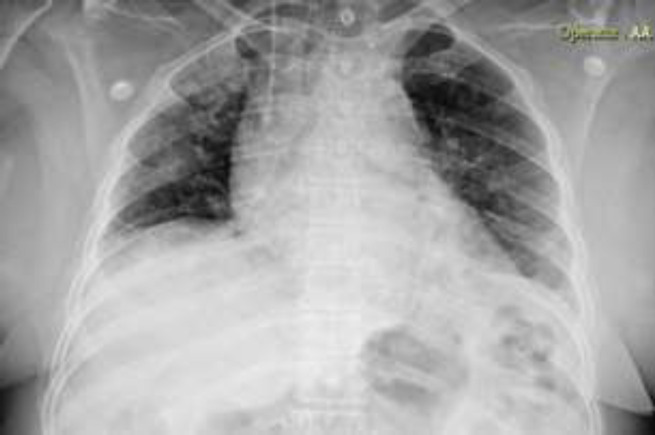
The lung infiltration resolved in the chest X-ray following the treatment

A 64-years-old female patient was admitted to the emergency department because of fever, dyspnea, and productive cough. The patient had a history of sarcoidosis diagnosed after cosmetic eyebrow tattooing and was receiving daily prednisolone (15-20mg) since the diagnosis and weakly methotrexate (10-15mg) 4 months ago. At the time of admission, the patient was febrile (37.8°C axillary) and had decreased oxygen saturation (89%). The rest of the physical examination was unremarkable except for a bilateral crackle on chest auscultation. By possible diagnosis of pneumonia, the patient received meropenem and ciprofloxacin. The laboratory results revealed a white blood cell count of 4400 (94% of neutrophil), platelet count of 163000, erythrocyte sedimentation rate of 57, creatinine level of 1.3, lactate dehydrogenase level of 800. According to the laboratory results and previous history of receiving prednisolone, a therapeutic dose of cotrimoxazole was added to the antimicrobial regimen to cover pneumocystis jirovecii infection. The primary chest x-ray imaging revealed bilateral infiltration, especially in the upper lobes and hilar lymphadenopathy. The lung high resolution computed tomography (HRCT) showed a bilateral diffuse nodular pattern (figure 1). After 72 hours of antimicrobial treatment, the fever was still present and the patient candidate for fiberoptic bronchoscopy. There was not any endobronchial lesion. Also, there was not any malignant cell or inclusion body reported. The gram staining of the bronchial fluid, polymerase chain reaction (PCR) for tuberculosis, and PCP was also unremarkable. However, the PCR for cytomegalovirus (CMV) was positive. While the PCR for PCP was negative, the cotrimoxazole was discontinued. The quantitative PCR for CMV form blood sample was taken and the result came back as 3.6*10^3^. With the impression of CMV pneumonia, a daily dose of 5mg of ganciclovir was prescribed. After 3 weeks of receiving 5mg/kg of ganciclovir twice daily (600mg daily), clinical symptoms, and dyspnea improved, likewise the radiological findings (figure 2). 

## Discussion

The present report demonstrated a case of CMV pneumonia in a patient who was a known case of sarcoidosis, receiving corticosteroids and cytotoxic drugs. The predisposing factor for sarcoidosis was cosmetic eyebrow tattooing. It has been demonstrated that chronic exposure to foreign materials including tattooing ink, exposes the immune system to systematized granulomatous hyperactivity (5). While most of the treatment regimens for sarcoidosis is based on reducing immune system activity, these patients may become susceptible to some infections. Baughman et al. reported that less than 1% of the patients with sarcoidosis develop fungal infections within the 18 months of follow-up (6). However, a similar study by Rubinstein et al. demonstrated that in over more than 7 years of follow -up, none of their patients developed an invasive opportunistic infection (7). 

The development of viral infections including CMV pneumonia is a rare complication in sarcoidosis patients receiving immunosuppressive drugs. Cunha et al. reported the first case of CMV infectious mononucleosis splenic infarct in a known case of sarcoidosis (3). They confirmed the diagnosis of CMV infectious mononucleosis with both liver and lung involvement by detecting elevated PCR viral load and IgM titer (3). CMV’s DNA amount varies across different patients. Immunosuppressed patients usually have higher viral DNA (4). 

In patients developing pneumonia, real-time PCR can detect actual CMV genome copy numbers in BAL fluid (4). Dureault et al. reported that the incidence of severe infection in sarcoidosis patients is 0.71% person-year (1). They also reported one probable CMV infection with dyspnea and infiltration of imaging studies as well as a positive PCR in BAL. Similar to our study, they treated their patient with ganciclovir (1). 

In the present report, we demonstrated that sarcoidosis patients receiving immunosuppressives are prone to develop CMV pneumonia, and fever and dyspnea were the alarm signs of CMV pneumonia is our patient which was successfully managed by ganciclovir. 

## Ethical Approval code:

The present article has been approved by Mashhad University of Sciences Ethics committee (IR.MUMS.MEDICAL.REC.1399.383).
